# Diagnostic validity of Autism Diagnostic Observation Schedule, second edition (K-ADOS-2) in the Korean population

**DOI:** 10.1186/s13229-022-00506-5

**Published:** 2022-06-30

**Authors:** So Yoon Kim, Miae Oh, Guiyoung Bong, Da-Yea Song, Nan-He Yoon, Joo Hyun Kim, Hee Jeong Yoo

**Affiliations:** 1 Teacher Education, Duksung Women’s University, Seoul, South Korea; 2 Department of Psychiatry, Kyung Hee University Hospital, Seoul, South Korea; 3 Department of Psychiatry, Seoul National University Bundang Hospital, Seoul National University College of Medicine, 300 Gumi-ro, Bundang-gu, Seongnam, Gyeonggi 463-707 South Korea; 4 Division of Social Welfare and Health Administration, Wonkwang University, Iksan, South Korea; 5 Seoul National University College of Medicine, Seoul, South Korea

**Keywords:** Autism, Diagnosis, Validity, ADOS-2

## Abstract

**Background:**

Although the Korean version of the Autism Diagnostic Observation Schedule-2 (K-ADOS‐2) is widely being used to diagnose autism spectrum disorder (ASD) in South Korea, no previous study has examined the validity and reliability of all modules of K-ADOS-2 across a wide age range, particularly older children, adolescents, and adults.

**Method:**

Data from 2,158 participants were included (mean age = 79.7 months; 73.6% male): 1473 participants with ASD and 685 participants without ASD (Toddler Module, *n* = 289; Module 1, *n* = 642; Module 2 *n* = 574; Module 3 *n* = 411; Module 4, *n* = 242). Participants completed a battery of tests, including the K-ADOS or K-ADOS-2 and other existing diagnostic instruments. Sensitivity, specificity, area under the receiver operating characteristic (ROC) curve, positive predictive value (PPV), negative predictive value (NPV), Cohen’s kappa (*k*), and agreement with existing diagnostic instruments were computed. Cronbach’s *α* values were also calculated.

**Results:**

All developmental cells of the K-ADOS-2 showed sufficient ranges of sensitivity 85.4–100.0%; specificity, 80.4–96.8%; area under the ROC curve, .90-.97; PPV, 77.8–99.3%; NPV, 80.6–100.0%; and *k* values, .83–.92. The kappa agreements of developmental cells with existing diagnostic instruments ranged from .20 to .90. Cronbach’s α values ranged from .82 to .91 across all developmental cells.

**Limitation:**

The best-estimate clinical diagnoses made in this study were not independent of the K-ADOS-2 scores. Some modules did not include balanced numbers of participants in terms of gender and diagnostic status.

**Conclusion:**

The K-ADOS-2 is a valid and reliable instrument in diagnosing ASD in South Korea. Future studies exploring the effectiveness of the K-ADOS-2 in capturing restricted, repetitive behaviors and differentiating ASD from other developmental disabilities are needed.

**Supplementary Information:**

The online version contains supplementary material available at 10.1186/s13229-022-00506-5.

## Introduction

Autism spectrum disorder (ASD) is a neurodevelopmental disorder characterized by social communication difficulties and the presence of repetitive and stereotyped behaviors, interests, and activities (RRBs) [1]. Due to the heterogeneity in symptom presentation of ASD, the clinical diagnosis is most valid and reliable when made using comprehensive diagnostic instruments [2, 3] such as *Autism Diagnostic Observation Schedule (*ADOS; [4]) or *ADOS-2* [5] and Autism Diagnostic Interview-Revised (ADI-R; [6]) [7, 8].

The ADOS-2 is a semi-structured, standardized observational instrument designed to assess and diagnose ASD across all ages. Initially developed in 1989 [9], the ADOS has been updated into the ADOS-2 to improve the accuracy and versatility of the assessment. The ADOS-2 revised classification algorithms, amended protocols of administration, included the additional module for toddlers between 12 and 30 months, and created new criteria for comparison scores, which allow the examination of ASD symptom severity across different modules [10]. The ADOS-2 classification of ASD requires an individual’s score to meet or exceed the algorithm threshold for the two domains: social affect and RRB. The schedule consists of five developmentally sequenced modules, each of which has a different combination of activities based on developmental age and expressive language skills. The wide usage of the ADOS-2 may be attributed to its ability to gather information from a set of structured activities, capture autistic behaviors during interactive activities, and account for the wide developmental levels and ages [11]. The ADOS-2 comprises social activities called “presses,” implemented to provide stimulating and standard contexts in which social communication behaviors and interactions are likely to appear [12].

The ADOS-2 has become more internationally accessible, driven by increased ASD awareness as well as the efforts to administer the ADOS-2 in different countries [13]. Currently, the ADOS-2 has been translated into more than 20 languages [14], and the clinical validity of the ADOS-2 has been well-established in various international samples [15]. Previous studies have underscored that ASD diagnostic instruments developed in Western countries can be properly translated and adapted in non-Western countries [13, 16–18]. Adapting the diagnostic tools that were originally developed for different cultures requires a re-examination of reliability and validity [7, 19]. Since culture influences the language, play materials, and social norms concerning developmentally appropriate behaviors, it can consequently affect how people in specific cultural contexts evaluate the appropriateness and severity of autistic symptoms [20, 21]. However, the majority of studies investigating the validity of translated versions of the ADOS-2 have been conducted in Western, English-speaking countries, such as the United States (US) [22], Canada [23], and the United Kingdom [24]. Only a handful of studies have examined the validity of the ADOS-2 in non-Western populations (e.g., in Chinese [16], Indian [17], and South Korean [13, 18]).

The Korean versions of the ADOS/ADOS-2 (i.e., K-ADOS/K-ADOS-2) have been used in South Korea for more than a decade [25]. To date, only two studies have partially validated the K-ADOS/K-ADOS-2 in South Korea. Kim et al. [18] conducted a study including 292 school students (aged 7–14 years old) to show that Module 3 of the K-ADOS had sufficient specificity and sensitivity. More recently, after the ADOS-2 was translated into Korean [26], Lee et al. [13] evaluated the validity of the Toddler Module and Modules 1 and 2 of the K-ADOS-2 on 143 South Korean toddlers and preschoolers. They found that the modules had adequate sensitivity, specificity, and internal consistency with respect to age. However, these previous studies were limited by their small sample size and relatively narrow age range of participants; therefore, research on the use and applicability of the K-ADOS-2, particularly on older children, adolescents, and adults, is still limited.

Further, researchers have emphasized the importance of establishing diagnostic utility of diagnostic instruments in differentiating ASD from other disabilities because ASD is often accompanied by and shows behavioral overlap with many neurodevelopmental and behavioral disorders (e.g., intellectual disabilities, anxiety disorders, and attention-deficit/hyperactivity disorders [ADHD]) [27–30], complicating the diagnostic process. Lee et al. [13] showed that the sensitivity and specificity of the K-ADOS-2 in distinguishing children and toddlers with ASD from those without ASD but have other developmental delays or language delays ranged from 94 to 100% and 82–100%, respectively. Yet, no other studies have examined the clinical validity of the K-ADOS-2 in distinguishing ASD from other developmental disabilities (OD), notably in Modules 3 and 4. It is particularly important to examine the diagnostic accuracy of the K-ADOS-2 in differentiating ASD from OD in adolescent and adult populations because the diagnostic process is considered more complicated due to increased comorbidities [31, 32]. Indeed, Langmann et al. [29] reported that the diagnostic validity of the Module 4 in distinguishing ASD from other clinical samples (e.g., personality disorders, behavioral and emotional disorders, anxiety and/or compulsive disorders) was low for older adults and individuals with high cognitive and verbal ability, suggesting the need for further research.

Therefore, the purpose of this study was to expand on previous findings [13, 18], examine the psychometric properties, and establish the diagnostic validity of the K-ADOS-2 across all modules (i.e., Toddler Module and Modules 1–4) with a larger number of participants. Specifically, we aimed to investigate (1) the diagnostic validity of all modules of the K-ADOS-2 algorithms, (2) its agreement with existing ASD diagnostic instruments, and (3) the reliability of all modules of the K-ADOS-2 to examine whether it can be validly and reliably applied to the South Korean population across all ages. Additionally, we preliminarily explored if the K-ADOS-2 could be used to differentiate ASD from OD.

## Methods

### Participants

This study is a secondary analysis of pooled data with research samples collected from 2008 to 2017 from several projects aimed at identifying ASD biomarkers, randomized control trials of social skills training, and developing an early ASD screening instrument. All the participants were enrolled via patient referrals from child and adolescent psychiatric, pediatric and child rehabilitation departments, and communities such as local clinics and daycare centers, recruitment posters on online/offline bulletin boards of public institutions, and online parenting communities. Participants from the social skills training programs consisted of participants with ASD; participants recruited for identification of ASD biomarkers and development of the early ASD screening instrument included both participants with ASD and without ASD. The examiners were blinded to the diagnostic characteristics of the participants, and clinical best-estimate diagnoses were determined by experienced clinicians, including two licensed child psychiatrists. One institution was in charge of recruiting participants and conducting all evaluations for all projects.

A total of 2158 participants were included in this study (mean age [standard deviation] = 79.7 [64.0] months; age range = 12–393 months; 1588 males; Toddler Module, *n* = 289; Module 1,^1^*n* = 642; Module 2 *n* = 574; Module 3 *n* = 411; Module 4, *n* = 242; 1473 participants with ASD, 685 participants without ASD, and 123 participants with OD). Participants with OD consisted of participants who were diagnosed as not having ASD based on clinical best-estimate diagnosis and obtained scores lower than 80 in either the full-scale intelligence quotients (FSIQ) or Korean Vineland Adaptive Behavior Scales, Second Edition (K-VABS; [33]) and therefore were considered as a subgroup of participants without ASD.

We aimed to categorize the OD group to represent individuals with *potential* intellectual disabilities or developmental delays. Although we were not able to confirm the clinical diagnostic status of the OD group, we wanted to, at least preliminarily, examine if the ADOS-2 can be used to differentiate individuals with ASD from individuals with at least some developmental problems in terms of adaptive skills and intellectual functioning. Diagnostic criteria of intellectual disability include deficits in intellectual and adaptive functionings observed during the developmental period [1], and, therefore, we used the FSIQs and K-VABS scores to identify individuals who may have an intellectual disability. We included participants with IQ scores lower than 80 to include those who have borderline intellectual functioning (i.e., individuals who function on the border between intellectual disability and normal intellectual functioning; [34]). Because the construct of adaptive behavior captures whether an individual has conceptual, social, practical skills expected of their age, development, and culture [35–37], we used the VABS score as a proxy for potential developmental delay.^2^

Diagnostic procedures are presented in the Procedures section. Detailed characteristics of the total participants and participants by module are included in Tables 1 and 2. Information on participant characteristics for each developmental cell of the Toddler Module, Module 1, and Module 2 is available in Additional file 1: Table S1. Detailed characteristics of the OD participants are available in Additional file 1: Table S2.

**Table 1:** Participant characteristics

Participant characteristics	Total		
	ASD mean (SD)	Non-ASD mean (SD)	*t*
Total *n*	1473	685	
% Male	82.3%	54.9%	
Age (months)	86.7 (65.9)	64.8 (56.9)	− 7.5**
FSIQ	84.5 (23.6)	101.6 (19.2)	9.9**
*ADOS-2*^a^			
SA	7.7 (1.7)	2.6 (1.8)	− 65.2**
RRB	5.7 (2.4)	1.6 (1.5)	− 48.7**
Total	7.1 (1.7)	1.9 (1.4)	− 76.0**
*ADI-R*			
SI	18.9 (6.3)	3.5 (3.5)	− 59.9**
C	14.0 (5.1)	2.0 (2.5)	− 49.6**
RRB	5.2 (2.6)	.8 (1.2)	− 42.6**
K-CARS	33.2 (5.4)	17.7 (3.3)	− 58.1**
SCQ	14.4 (7.1)	4.4 (5.1)	− 26.0**
SRS	86.6 (29.8)	36.8 (21.5)	− 36.6**

*Notes* *p < .05, **p < .01. ASD, autism spectrum disorder; FSIQ, full-scale intelligence quotient; ADOS-2, Autism Diagnostic Observation Schedule-2; SA, social affect; RRB, Restricted Repetitive Behavior; ADI-R, Autism Diagnostic Interview-Revised; SI, social interaction; C, Communication Total; RRB, Restricted Repetitive; K-CARS, Korean Child Autism Rating Scale; SCQ, Social Communication Questionnaire; and SRS, Social Responsiveness Scale. ^a^ = reported in calibrated severity scores

**Table 2:** Participant characteristics by module

Participant characteristics	Toddler Module			Module 1			Module 2			Module 3			Module 4		
	ASD mean (SD)	Non-ASD mean (SD)	*t*	ASD mean (SD)	Non-ASD mean (SD)	*t*	ASD mean (SD)	Non-ASD mean (SD)	*t*	ASD mean (SD)	Non-ASD mean (SD)	*t*	ASD mean (SD)	Non-ASD mean (SD)	*t*
*n*	96	193		564	78		388	186		233	178		192	50	
% Male (%)	70.8	56.5		80.1	65.4		80.4	60.8		86.3	47.2		93.2	38.0	
Age (months)	24.9 (4.4)	21.6 (5.2)	− 5.4**	52.5 (21.9)	37.8 (19.5)	− 5.6**	66.3 (30.2)	43.8 (11.8)	− 9.8**	116.5 (32.9)	105.8 (33.8)	− 3.2**	223.0 (53.8)	205.6 (58.0)	− 2.0*
FSIQ	NA	NA	NA	60.4 (17.5)	97.0 (28.6)	3.4**	72.1 (21.4)	92.4 (18.8)	5.6**	89.2 (21.3)	103.8 (18.0)	6.8**	97.5 (18.4)	103.3 (21.1)	1.8
ADOS-2 ^a^															
SA	7.9 (1.8)	2.5 (1.6)	− 24.7**	7.4 (1.7)	2.6 (1.5)	− 25.2**	7.6 (1.5)	2.5 (1.6)	− 37.2**	8.2 (1.4)	2.9 (2.2)	− 28.1**	7.7 (1.6)	1.9 (1.7)	− 22.3**
RRB	4.9 (2.4)	1.8 (1.6)	− 11.5**	6.1 (2.2)	1.9 (1.8)	− 15.4**	5.7 (2.3)	1.9 (1.6)	− 22.8**	5.0 (3.0)	1.2 (.7)	− 19.2**	5.9 (1.9)	1.3 (1.2)	− 21.4**
Total	7.0 (1.8)	2.0 (1.3)	− 24.6**	6.9 (1.6)	2.0 (1.4)	− 24.6**	7.0 (1.5)	1.8 (1.1)	− 46.2**	7.5 (1.7)	2.1 (1.7)	− 32.0**	7.3 (1.7)	1.5 (1.1)	− 29.8**
ADI-R															
SI	15.1 (5.0)	4.2 (3.2)	− 22.5**	19.5 (6.2)	5.8 (4.3)	− 19.2**	18.2 (6.0)	2.7 (2.7)	− 33.6**	19.1 (6.5)	2.9 (3.6)	− 29.6**	20.1 (6.5)	1.9 (2.9)	− 18.9**
C	6.2 (7.9)	1.3 (1.7)	− 4.0**	12.3 (5.1)	3.7 (3.2)	− 10.9**	14.8 (4.4)	1.8 (1.9)	− 37.8**	14.4 (5.2)	2.0 (2.8)	− 28.3**	15.2 (5.1)	1.5 (2.4)	− 18.2**
RRB	3.5 (2.2)	.9 (1.1)	− 13.6**	4.8 (2.2)	1.3 (1.5)	− 13.8**	5.8 (2.8)	.9 (1.3)	− 22.7**	5.8 (2.7)	.5 (.9)	− 24.7**	5.3 (2.6)	.6 (1.2)	− 12.5**
K-CARS	31.6 (3.5)	17.5 (3.5)	− 27.7**	34.5 (5.8)	19.0 (3.3)	− 20.6**	32.7 (5.0)	17.3 (2.8)	− 33.3**	31.7 (5.0)	18.2 (3.4)	− 23.6**	32.1 (4.0)	17.1 (2.5)	− 12.1**
SCQ	15.2 (4.5)	7.9 (5.3)	− 8.2**	17.0 (6.5)	6.0 (5.5)	− 9.7**	13.7 (7.0)	3.1 (3.6)	− 14.8**	11.5 (7.3)	3.4 (5.3)	− 11.6**	12.9 (7.2)	3.9 (4.4)	− 7.8**
SRS	64.5 (16.3)	48.1 (8.1)	− 10.4**	89.6 (30.5)	46.0 (19.8)	− 10.9**	82.8 (28.8)	35.3 (19.4)	− 19.6**	89.1 (30.3)	24.8 (22.4)	− 22.6**	93.7 (28.9)	31.0 (33.8)	− 12.2**

*Notes* *p < .05, **p < .01. ASD, autism spectrum disorder; FSIQ, full-scale intelligence quotient; ADOS-2, Autism Diagnostic Observation Schedule-2; SA, social affect; RRB, Restricted Repetitive Behavior; ADI-R, Autism Diagnostic Interview-Revised; SI, social interaction; C, Communication Total; RRB, Restricted Repetitive; K-CARS, Korean Child Autism Rating Scale; SCQ, Social Communication Questionnaire; and SRS, Social Responsiveness Scale. ^a^ = reported in calibrated severity scores

### Procedures

Participants and their parents completed a battery of tests during their one-time visit, including the K-ADOS or K-ADOS-2, ADI-R, the Korean version of Childhood Autism Rating Scale (K-CARS), Korean Vineland Social Maturity Scale (K-SMS), and cognitive tests measuring FSIQs. Questionnaires, such as the Social Responsiveness Scale-2 (SRS-2), Social Communication Questionnaire (SCQ), and K-VABS, were mailed and filled out prior to the visit. The K-ADOS or K-ADOS-2 and ADI-R were administered by research-reliable professionals or research assistants who worked alongside them in the same laboratory on a daily basis and were trained prior to the actual administration. The scales were administered only after an adequate level of inter-reliability with the research-reliable professionals (> 80%) was reached. All administrations of the K-ADOS or K-ADOS-2 and ADI-R were videotaped and double-checked by these professionals to confirm the quality and reliability.

Subsequently, two board-certified psychiatrists made the best-estimate clinical diagnostic criteria for ASD and non-ASD based on DSM-5 [1]. The clinical best-estimate diagnosis was made according to the information gathered collectively from all tests administered, including the K-ADOS/K-ADOS-2, ADI-R, SCQ_,_ SRS-2, K-CARS, SMS, VABS, IQ assessments, and observed clinical impressions. The study was approved by the Institutional Review Board (IRB) of Seoul National University Bundang Hospital (IRB no. B-2110–716-102).

### Measures

#### Autism Diagnostic Observation Schedule and Autism Diagnostic Observation Schedule-2 (ADOS and ADOS-2 [4, 5])

This study used the Korean translated versions of the ADOS/ADOS-2, approved by its publisher Western Psychological Services. Data collected prior to July 2017, when the ADOS-2 was published in Korea, were administered using the original K-ADOS. The results from the K-ADOS were rescored based on the K-ADOS-2 algorithm for this study. The modules range from the Toddler Module, for children aged 30 months and younger, to Module 4, for verbally fluent older adolescents and adults. The diagnostic algorithms for the Toddler Module and Modules 1 and 2 are further subdivided into developmental cells based on age/language. The algorithm for the Toddler Module is divided into two developmental cells: 12–20 months/nonverbal 21–30 months toddlers (12–20/NV21–30) and 21–30 months toddlers with some words (21–30SW). The algorithm of Module 1 is divided into two developmental cells based on expressive language level: no words (NW) and some words (SW). The algorithm of Module 2 is divided into two developmental cells based on age groups: < 5 years and ≥ 5 years.

All modules provide two cutoff points in the classification algorithms. For Modules 1 through 4, there is a higher cutoff in the classification algorithms for stringent classification (i.e., *autism*) and a lower cutoff in the classification algorithms for more inclusive classification (that is, autism spectrum disorder; *ASD*). For Module 4, we applied the revised algorithm from Hus and Lord [38]. The Toddler Module also has a higher cutoff in the classification algorithms for stringent classification (*moderate–severe concern*) and a lower cutoff in the classification algorithms for more inclusive classification (*mild–moderate concern),* which were specified in Esler et al. [39]. Alternatively, Luyster et al. [40] provided the single research cutoff point for the Toddler Module and explained that the single cutoff needs to be applied in the Toddler Module due to the relative lack of diagnostic stability in younger children. In this study, we primarily relied on the results calculated based on the *ASD cutoff* for Modules 1–4 and the Luyster et al. [40]’s cutoff point for Toddler Module to make the decisions regarding validity. The diagnostic validity of the Toddler Module calculated based on Esler et al. [39]’s cutoff point system is presented in Additional file 1: Table S3.

#### Autism Diagnostic Interview-Revised (ADI-R [6])

The ADI-R is a semi-structured caregivers’ interview used to diagnose or evaluate the core symptoms of ASD. Each item is scored and converted on a scale of 0, 1, and 2, with higher scores indicating a greater number of and/or clear symptoms of ASD. The ADI-R includes 93 items describing four diagnostic domains: social interaction, communication, RRBs, and abnormality of development evident at or before 36 months. Each domain has a diagnostic criterion, but individuals must exceed all four cutoff scores to be classified as ASD. While the majority of the algorithm score consists of parents’ descriptions of a child’s behaviors between the ages of 4–5 years, some items ask whether the behavior has ever been present during the child's lifetime. For children under 4 years of age, ratings on current behaviors are used. The Korean translation of the ADI-R [25], approved by its publisher Western Psychological Services, was used in this study.

#### Social Communication Questionnaire [41]

The SCQ is a caregiver-report screening instrument for ASD designed to evaluate an individual’s behavior in three domains: social interaction, language and communication, and RRB. The SCQ includes 40 items to be rated as either “yes” or “no.” It consists of two forms: the Lifetime Form, which focuses on an individual’s developmental history, and the Current Form, which inspects an individual’s behaviors over the past three months. The total score in the Lifetime Form is used to determine if an individual is likely to have ASD, and whether a more extended diagnostic evaluation needs to be undertaken. In this study, we used a cutoff score of 10, for children under 47 months of age, and 12, for children over 48 months, based on a standardization study conducted in Korea [42].

#### Social Responsiveness Scale-2 (SRS-2 [43])

The SRS-2 is a 65-item parent-report questionnaire that assesses the severity of ASD-related symptoms on a 4-point scale, with higher total scores reflecting more severe ASD symptomatology. It consists of five subscales: social awareness, social cognition, social communication, social motivation, and autistic mannerisms. The SRS-2 has been used extensively in the ASD literature as a diagnostic measure [44] and is reported to have good internal consistency and concurrent, discriminant validity [45]. Chun et al. [46] demonstrated adequate levels of sensitivity and specificity of the Korean translated version of the SRS-2. A cutoff T-score of 65 was applied regardless of gender in the preschool form of the SRS-2, and cutoff T-scores of 70 and 63 were used for female and male participants, respectively, for the school-age and adult forms of the SRS-2 because these values are widely used across clinical settings in South Korea.

#### Korean version of the Childhood Autism Rating Scale (K-CARS [47])

The CARS [48] is a clinician-rated scale developed to screen for ASD. Consisting of 15 items rating the presence and severity of symptoms associated with ASD, the CARS is scored from 1 (no impairment observed or reported) to 4 (severe impairment). There is no consensus on the cutoff score of the K-CARS; Shin and Kim [49] suggested a cutoff score of 28, while others recommend 24 [50]. Therefore, we utilized both cutoff scores in this study.

#### Full-Scale Intelligence Quotients (FSIQ)

The following instruments were used to calculate FSIQ in this study: the Wechsler Preschool and Primary Scale of Intelligence (WPPSI) [51] for children aged 2 years and 6 months to 6 years, Wechsler Intelligence Scale for Children (WISC) [52] for children aged 6–16 years, and Wechsler Adult Intelligence Scale (WAIS) [53] for individuals over 16 years of age. These instruments utilize chronological age standardization with a mean of 100 and a standard deviation of 15.

#### Korean version of the Vineland Adaptive Behavior Scale, second edition (K-VABS [33, 54])

The VABS is a parent or other caregiver’s rating of a person’s adaptive functioning and social self-sufficiency from birth to adulthood. The VABS consists of five domains: communication, daily living skills, socialization, motor skills, and maladaptive behavior. It is scored on a 0–2 rating scale, with a higher score representing skills used more frequently. The five domains together yield a total adaptive behavior composite score. The normative mean of the composite score is 100, with a standard deviation of 15. We used the Korean version of the parent/caregiver rating form of VABS, which was highly correlated with the survey interview form of VABS and showed sufficient validity among Koreans [55].

#### Korean Vineland Social Maturity Scale (K-SMS [56])

The K-SMS is a clinician-rated instrument that assesses social and adaptive maturity. Originally developed using the Doll’s Vineland Social Maturity Scale [57], the K-SMS includes 89 items grouped by behavioral milestones that are expected at each age. It consists of eight subdomains (communication, general self-help, locomotion, occupation, self-direction, self-help eating, self-help dressing, and socialization skills) and provides a global social age and social quotient.

#### Nonverbal mental age

Data were collected from multiple studies aiming to fulfill different objectives; the age range of participants recruited for each study and, consequently, the scales used to assess the nonverbal mental age of participants varied across studies. Depending on the type and age range of the studies, we used the Beery-Buktenica Developmental Test of Visual-Motor Integration (VMI) or Leiter International Performance Scale in addition to the nonverbal subscale of WPPSI or WISC. The Beery-Buktenica Developmental Test measures the ability of an individual to integrate their visual perception and motor coordination [58]. The Leiter International Performance Scale assesses nonverbal performance intelligence and cognitive abilities [59]. Many participants were not able to participate in these assessments of nonverbal mental age due to lack of cooperation, and, additionally, some could not participate because they did not meet the minimum age range for participation. For instance, we could not collect the information about the nonverbal mental age of participants in the Toddler Module. However, we present the information on the nonverbal mental age of participants in Module 1, analyzed using the collected data, since Gotham et al. [22] reported that the specificity was low when Module 1 was applied to children with nonverbal mental age lower than 15 months.

We identified the nonverbal mental age of 30 participants in Module 1, calculated based on the WPPSI or WISC scores, and, of these 30 participants, none of the participants in Module 1 had a nonverbal mental age lower than 15 months. We also identified the VMI scores from 74 participants of the participants in Module 1, and the developmental age calculated based on the VMI scores of all 74 participants exceeded 35 months (mean developmental age = 43.4 months, SD = 10.4). Additionally, we identified the Leiter International Performance Scale of 169 participants in Module 1, and five participants with ASD from Module 1 had a nonverbal mental age lower than 15 months. We conducted sensitivity tests of the entire analysis on Module 1, Module 1 SW, and Module 1 NW after eliminating these five participants, and eliminating these participants resulted in very minimal changes in analyses.

### Statistical analyses

Initially, we computed a set of independent t tests comparing the age, FSIQ, and scores from K-ADOS-2, ADI-R, K-CARS, SCQ, and SRS-2 of participants with ASD and those without ASD. Calibrated severity scores (CSS; i.e., a severity metric that takes age and language level into account [60] were used to compare the K-ADOS-2 scores.

To address the first aim, the sensitivity, specificity, PPV, NPV, and Cohen’s kappa (*k*) between ASD and non-ASD were calculated to check for consistency between the best-estimate clinical diagnosis and diagnosis based on *ASD cutoff* for K-ADOS-2 Modules 1–4 and Luyster et al.’s [40] cutoff point for Toddler Module. This analysis was conducted on all modules combined, each module (including Toddler Module and Modules 1, 2, 3, and 4) individually, and each developmental cell (12–20/NV21–30 and 21–30 SW in Toddler Module, NW and SW in Module 1, and under and over 5 years of age in Module 2). We also computed the area under the receiver operating characteristic (ROC) curve of all items by developmental cell to explore if all items included in the algorithm have sufficient diagnostic accuracy according to the area under the curve (AUC).

To investigate the second aim, we computed Pearson’s r correlation coefficients between the total scores of K-ADOS-2 and those of existing ASD diagnostic instruments (i.e., ADI-R, K-CARS, SCQ, and SRS-2) for all modules combined, each module individually, and each developmental cell. Additionally, *k* values were calculated between the diagnosis based on the K-ADOS-2 *ASD cutoff* (and Luyster et al.’s [40] cutoff point for Toddler Module) and the diagnosis based on the existing ASD diagnostic instruments. The *k* values were interpreted based on McHugh’s [61] criteria (0–0.2, none; 0.21–0.39, minimal; 0.40–0.59, weak; 0.60–0.79, moderate; 0.8–0.9, strong; above 0.9, almost perfect). For the third aim, Cronbach’s α values for the algorithm items and values after an item was removed were computed to examine the internal consistency of each developmental cell.

Finally, we calculated the sensitivity, specificity, PPV, NPV, and *k* values to examine how accurately the K-ADOS-2 *ASD cutoff* can distinguish ASD from OD for all modules combined, each module individually, and each developmental cell. We did not compare the diagnostic validity between OD and the remaining participants without ASD (i.e., participants who were not diagnosed with ASD and did not have FSIQ or VABS scores lower than 80) because this sample included a few participants for whom we did not have all FSIQ and VABS scores and therefore would have been categorized as OD if all relevant information was available.

All analyses except for the calculation of Cronbach’s α values were repeated using the *Autism cutoff* for Modules 1–4 and *moderate–severe concern* for the Toddler Module. All statistical analyses were performed using Excel and SPSS Statistics (version 23.0; IBM Corp., Armonk, NY, USA).

## Results

There were statistically significant inter-group differences between ASD and non-ASD in all algorithm scores of the K-ADOS-2, ADI-R, K-CARS, SCQ, and SRS-2 (*p* < 0.01) in the composite K-ADOS-2 and across all developmental cells, except for in the ADI-R Communication domain in the 12–20/NV21–30 developmental cell group (Tables 1, 2 and Additional file 1: Table S1).

All developmental cells of the K-ADOS-2 showed sufficient ranges of sensitivity 85.4–100.0%; specificity, 80.4–96.8%; area under the ROC curve, 0.90–0.97; PPV, 77.8–99.3%; NPV, 80.6–100.0%; and *k* values, 0.83–0.92.^3^ Detailed results of the sensitivity, sensitivity, AUC, PPV, NPV, and *k* values between ASD versus non-ASD by module and developmental cell are presented in Table 3.

**Table 3:** Sensitivity, specificity, AUC, PPV, NPV, and Cohen’s kappa between ASD and non-ASD based on ASD cutoff criteria

Module	*n*	Sensitivity (%)	Specificity (%)	AUC	PPV (%)	NPV (%)	Cohen’s kappa (*p*-value)
Total Modules	2158	98.1	90.5	.94	95.7	95.7	.89 (*p* < .001)
Toddler Module	289	87.5	95.9	.92	91.3	93.9	.84 (*p* < .001)
Toddler Module (12–20/NV21–30)	206	85.4	96.8	.91	94.6	90.9	.84 (*p* < .001)
Toddler Module (21–30 SW)	83	100.0	94.2	.97	77.8	100.0	.85 (*p* < .001)
Module 1	642	98.2	84.6	.91	97.9	86.8	.84 (*p* < .001)
Module 1 (NW)	314	97.9	92.6	.95	99.3	80.6	.85 (*p* < .001)
Module 1 (SW)	328	98.6	80.4	.90	96.5	91.1	.83 (*p* < .001)
Module 2	574	99.2	91.4	.95	96.0	98.3	.92 (*p* < .001)
Module 2 (< 5 yo)	350	98.9	92.7	.96	93.9	98.7	.92 (*p* < .001)
Module 2 (≥ 5 yo)	224	99.5	81.8	.91	98.0	94.7	.87 (*p* < .001)
Module 3	411	99.6	87.1	.93	91.0	99.4	.88 (*p* < .001)
Module 4	242	99.0	88.0	.94	96.9	95.7	.90 (*p* < .001)

*Notes* AUC, Area under curve; PPV, positive predictive value; NPV, negative predictive value; ASD, autism spectrum disorder; 12–20/NV21–30, 12–20 months toddlers/nonverbal 21–30 months toddlers; 21–30 SW, 21–30 months toddlers with some words; NW, no words; SW, some words; and yo, years old

The AUC values of the majority of algorithm items in each developmental cell exceeded 0.70 (range = 0.70–0.93). The list of algorithm items with AUC values lower than 0.70 is presented in Table 4 by developmental cell. Across all developmental cells, the AUCs of *Hand Finger and Other Complex Mechanism item* were consistently lower than 0.70, and all items with AUC lower than 0.7 were from the RRB algorithm.

**Table 4:** Algorithm items with AUC values lower than .7

Module	Item #	Item description	AUC value
Toddler Module (12–20/NV21–30)	A3	Intonation of vocalization or verbalizations	.60
	D1	Unusual sensory interest in play material/person	.65
	D2	Hand and finger movements/posturing	.56
	D5	Unusually repetitive interests or stereotyped behaviors	.68
Toddler Module (21–30 SW)	D2	Hand and finger movements/posturing	.54
Module 1 (NW)	D2	Hand and finger and other complex mannerisms	.63
Module 1 (SW)	D2	Hand and finger and other complex mannerisms	.61
Module 2 (< 5 yo)	D1	Unusual sensory interest in play material/person	.60
Module 2 (≥ 5 yo)	D2	Hand and finger and other complex mannerisms	.60
Module 3	A4	Stereotyped/idiosyncratic use of words or phrases	.69
	D1	Unusual sensory interest in play material/person	.61
	D2	Hand and finger and other complex mannerisms	.55
Module 4	A4	Stereotyped/idiosyncratic use of words or phrases	.60
	D1	Unusual sensory interest in play material/person	.52
	D2	Hand and finger and other complex mannerisms	.53
	D4	Excessive interest in or references to unusual or highly specific topics or objects or repetitive behaviors	.67

*Notes* 12–20/NV21–30, 12–20 months toddlers/nonverbal 21–30 months toddlers; 21–30 SW, 21–30 months toddlers with some words; NW, no words; SW, some words; yo, years old; and AUC, area under curve

The total scores of the K-ADOS-2 were significantly and positively correlated with those of ADI-R, SCQ, SRS-2, and K-CARS scores across all modules and developmental cells. Pearson’ r correlations ranged between 0.60 and 0.90 for Toddler Module (12–20/NV21–30), 0.57–0.90 for Toddler Module (21‐30SW), 0.45–0.80 for Module 1 (NW), 0.54–0.78 for Module 1 (SW), 0.66–0.88 for Module 2 (< 5 yo), 0.55–0.68 for Module 2 (≥ 5 yo), 0.52–0.82 for Module 3, and 0.47–0.84 for Module 4. The kappa agreements between all K-ADOS-2 modules and existing diagnostic instruments ranged between 0.48–0.85 for Toddler Module (12–20/NV21–30), 0.47–0.90 for Toddler Module (21‐30SW), 0.35–0.82 for Module 1 (NW), 0.38–0.64 for Module 1 (SW), 0.54–0.72 for Module 2 (< 5 yo), 0.20–0.42 for Module 2 (≥ 5 yo), 0.33–0.73 for Module 3, and 0.25–0.57 for Module 4, suggesting weak-to-strong agreement. Detailed results of Pearson's correlations and kappa values with existing diagnostic instruments by module and developmental cell are presented in Table 5.

**Table 5:** Agreement with existing instrument based on ASD cutoff criteria

Module	Pearson’s r (Cohen’s Kappa values)				
	ADI-R	SCQ	SRS	K-CARS (cutoff 24)	K-CARS (cutoff 28)
Total Modules	.77**	.62**	.63**	.86**	
	(.66**)	(.52**)	(.61**)	(.69**)	(.59**)
Toddler Module	.75**	.62**	.62**	.90**	
	(.58**)	(.49**)	(.57**)	(.86**)	(.83**)
Toddler module (12–20/NV21–30)	.89**	.62**	.60**	.90**	
	(.59**)	(.48**)	(.58**)	(.85**)	(.83**)
Toddler module (21–30 SW)	.85**	.57**	.59**	.90**	
	(.49**)	(.47**)	(.51**)	(.90**)	(.81**)
Module 1	.70**	.52**	.55**	.79**	
	(.41**)	(.40**)	(.42**)	(.71**)	(.53**)
Module 1 (NW)	.52**	.47**	.45**	.80**	
	(.35**)	(.42**)	(.35**)	(.82**)	(.65**)
Module 1 (SW)	.78**	.54**	.65**	.76**	
	(.45**)	(.38**)	(.47**)	(.64**)	(.45**)
Module 2	.82**	.64**	.66**	.87**	
	(.68**)	(.51**)	(.58**)	(.69**)	(.58**)
Module 2 (< 5 yo)	.85**	.66**	.66**	.88**	
	(.72**)	(.54**)	(.61**)	(.69**)	(.59**)
Module 2 (≥ 5 yo)	.66**	.58**	.55**	.68**	
	(.42**)	(.28**)	(.31**)	(.38**)	(.20*)
Module 3	.78**	.52**	.64**	.82**	
	(.73**)	(.50**)	(.61**)	(.44**)	(.33**)
Module 4	.74**	.47**	.59**	.84**	
	(.57**)	(.44**)	(.55**)	(.28**)	(.25**)

Notes **p-value 1 < 0.001; *p-value < .01; ASD, autism spectrum disorder; 12–20/NV21–30 12–20 months toddlers/nonverbal 21–30 months toddlers; 21–30 SW, 21–30 months toddlers with some words; NW, no words; SW, some words; ADI-R, Autism Diagnostic Interview-Revised; SCQ, Social Communication Questionnaire; SRS, Social Responsiveness Scale; and K-CARS, Korean Child Autism Rating Scale

All modules and developmental cells had high internal consistencies, with α values ranging from 0.82 to 0.91. Removing an item inflicted no-to-minimal changes (that is, a change of less than 0.03 change in α values). The complete results of the reliability analysis are presented in Table 6.

**Table 6:** Results of reliability analysis

Reliability	Toddler (12–20/NV21–30)	Toddler (21–30 SW)	Module 1 (No Words)	Module 1 (Words)	Module 2	Module 3	Module 4
Cronbach’s alpha	.91	.85	.82	.86	.83	.88	.89
Item discrimination (range)	.89–.91	.83–.85	.79–.82	.83–.86	.81–.83	.87–.88	.87–.89

Notes. 12–20/NV21–30, 12–20 months toddlers/nonverbal 21–30 months toddlers; 21–30 SW, 21–30 months toddlers with some words; NW, no words; SW, some words; and yo, years old

There were no significant differences in participants’ age between the OD and ASD groups except for in Module 2 (OD, *M* = 50.2 months; ASD, *M* = 66.3 months; *p* = 0.01). The IQ scores of the OD and ASD groups only differed significantly in Module 4 (OD, *M* = 73.0; ASD, *M* = 97.5; *p* = 0.0001). There were statistically significant group differences in OD vs. ASD in all algorithm scores of the K-ADOS-2 and ADI-R (*p*s < 0.05) across all developmental cells (Additional file 1: Table S2). When using the *ASD cutoff* to distinguish OD from ASD*,* all modules and developmental cells of the K-ADOS-2 had sufficient sensitivity, specificity, AUC, PPV, and NPV except for NPV in Toddler Module and Module 1. Sensitivity across the developmental cells ranged from 85.4 to 100.0%; specificity, 66.7–94.7%; AUC, 0.83-0.97; PPV, 93.3–99.5%; and NPV, 50.0–100% (Table 7). The *k* values with the final diagnosis ranged between 0.53 and 0.92, suggesting moderate-to-almost perfect agreement based on McHugh’s [61] criteria.

**Table 7:** Sensitivity, specificity, AUC, PPV, NPV, and Cohen’s kappa between ASD and OD based on ASD cutoff criteria

Module	N of ASD	N of OD	Sensitivity (%)	Specificity (%)	AUC	PPV (%)	NPV (%)	Cohen’s kappa (*p*-value)
Total	1473	123	98.1	79.7	.89	98.3	77.8	.77** (*p* < .001)
Toddler Module	96	22	87.5	81.8	.85	95.5	60.0	.61** (*p* < .001)
Toddler Module (12–20/NV21–30)	82	15	85.4	80.0	.83	95.9	50.0	.53** (*p* < .001)
Toddler Module (21–30 SW)	14	7	100.0	85.7	.93	93.3	100.0	.89** (*p* < .001)
Module 1	564	27	98.2	81.5	.90	99.1	68.8	.73** (*p* < *.*001)
Module 1 (NW)	287	12	97.9	83.3	.91	99.3	62.5	.70** (*p* < .001)
Module 1 (SW)	277	15	98.6	80.0	.89	98.9	75.0	.76** (*p* < .001)
Module 2	388	26	99.2	92.3	.96	99.5	88.9	.90** (*p* < .001)
Module 2 (< 5 yo)	186	19	98.9	94.7	.97	99.5	90.0	.92** (*p* < .001)
Module 2 (≥ 5 yo)	202	7	99.5	85.7	.93	99.5	85.7	.85** (*p* < .001)
Module 3	233	39	99.6	71.8	.86	95.5	96.6	.80** (*p* < .001)
Module 4	192	9	99.0	66.7	.83	98.4	75.0	.70** (*p* < .001)

Notes. *p < .05, **p < .01. AUC, area under curve; PPV, positive predictive value; NPV, negative predictive value; ASD, autism spectrum disorder; OD, other developmental disabilities; 12–20/NV21–30 12–20 months toddlers/nonverbal 21–30 months toddlers; 21–30 SW, 21–30 months toddlers with some words; NW, no words; SW, some words; and yo, years old

Additional file 1: Tables S4 and S5 present the sensitivity, specificity, AUC, PPV, NPV, and *k* values between ASD and non-ASD and those between ASD and OD, respectively, calculated based on the *Autism cutoff* score for Modules 1–4 and *moderate–severe concern* range for Toddler Module. Additional file 1: Table S6 presents the agreement with existing diagnostic instruments, calculated based on the *Autism cutoff* score for Modules 1–4 and *moderate–severe concern* range for Toddler Module*.*

## Discussion

This study showed that the K-ADOS-2 has excellent diagnostic validity in distinguishing individuals with ASD from those without ASD with sufficiently high sensitivity, specificity, AUC, PPV, and NPV across a wide age group. Moreover, all modules and developmental cells of the K-ADOS-2 demonstrated sufficient reliability. These findings provide additional evidence that the ADOS-2 can be adapted for various cultural settings [7, 13, 16, 62]. This suggests that although there can be cultural differences in the interpretation of severity and appropriateness of autistic behaviors [20, 21], the behavior patterns that need to be considered when diagnosing ASD may not differ across cultures.

Further, compared to previous adaptation studies conducted in different countries such as the Netherlands [62] and Poland [7], the K-ADOS-2 exhibited higher sensitivity and specificity values. As Lee et al. [13] also highlighted the importance of highly trained, research-reliable clinicians in establishing strong validity and specificity for a measure, we postulate that the positive result from this study may be due to the strict, reliable administration and coding process in which research-reliable professionals double-checked all K-ADOS-2 administration.

The examination of the AUC under each item indicated that all algorithm items in the social affect domain had an acceptable ability to distinguish ASD from non-ASD. Meanwhile, the *Hand Finger and Other Complex Mechanism item* showed consistently low AUC across all developmental cells*,* and items with AUC lower than 0.7 were all from the RRB algorithm. Similarly, previous studies have also suggested that social communicational items tend to distinguish the individuals with ASD from those without ASD more accurately than the RRB items [63–65]. Given the brevity of the time allotted for the observation during the K-ADOS-2 and the variability of frequency and types of RRBs depending on the observational contexts (i.e., clinic vs. home) [66], it is possible that clinicians are not offered a sufficient opportunity to observe these types of RRBs during the K-ADOS-2. We, therefore, suggest the importance of complementing the results of K-ADOS-2 with other diagnostic instruments such as the ADI-R that rely on a more long-term observation by parents or teachers, particularly when assessing the RRBs.

The K-ADOS-2 scores were correlated with the ADI-R, SRS-2, SCQ, and K-CARS scores across all developmental cells and modules, suggesting sufficient concurrent validity. Interestingly, the Pearson’s r coefficients between K-ADOS-2 and ADI-R and K-CARS tended to be greater than those between K-ADOS-2 and SCQ and SRS-2. This pattern could be explained by the inherent shortcomings of parent-report questionnaires (i.e., the SRS-2 and SCQ). Caregivers may have responded to the questions based on their interpretations without an accurate understanding of the concepts captured in each question [67]. Caregivers’ beliefs, characteristics, acceptance, and awareness of ASD may have influenced how they interpreted their child’s behaviors [68, 69].

It is noteworthy that kappa agreements between diagnoses made by the K-ADOS-2 and SCQ, SRS-2, and K-CARS were weak in some modules and developmental cells. In particular, the kappa agreements between K-ADOS-2 and K-CARS were low in ≥ 5 yo developmental cell of Module 2 and Modules 3 and 4. However, considering that the Pearson’s correlations between them were strong and significant, we postulate that this discrepancy may signal the need for more studies adjusting cutoff scores on the K-CARS in the Korean population, especially for the verbally fluent children, adolescents, and adults with ASD. Indeed, due to the lack of consistency in the K-CARS cutoff score used in Korea, we applied two cutoff scores (i.e., [21, 41]) [49, 50] to calculate agreement with K-ADOS-2 scores.

Similar to the findings from the previous K-ADOS-2 adaptation study of the Toddler Module and Modules 1 and 2 [13], applying an *autism* (i.e., higher) cutoff lowered the sensitivity and specificity compared to using an *ASD* (i.e., lower) cutoff. However, previous validation studies of the ADOS-2 conducted in Western countries such as the US [70] and Sweden [71] have reported more balanced specificities and sensitivities when applying *an autism cutoff,* suggesting that sample variability may impact the diagnostic validity of the ADOS-2 [13].

In our preliminary examination of the K-ADOS-2’s validity in differentiating ASD from OD, we found promising results that sensitivity, specificity, AUC, PPV, and Cohen’s kappa were satisfactory for all developmental cells. However, NPV values of Toddler Module and Module 1, particularly the 12–20/NV21–30 algorithm of Toddler Module and the NW algorithm of Module 1, were relatively low. This suggests that children with developmental difficulties, especially those who do not use words to communicate, should be examined with additional diagnostic instruments even if the K-ADOS-2 identifies them as non-ASD. Notably, however, it is unclear if some of the participants categorized as having OD in this study had a formal developmental disability diagnosis. Different patterns could have emerged if we had included individuals with a confirmed diagnosis of non-ASD developmental disabilities (e.g., intellectual disabilities) as a separate clinical control group, and future studies should investigate this possibility.

### Limitations

This study has several limitations, which recommend promising avenues for future studies. First, while the best-estimate clinical diagnosis was based on the combination of direct observation, caregiver reports, and other psychological assessments, the final diagnosis was not independent of the K-ADOS-2 scores. To establish its validity more accurately, we suggest that separate institutions independently implement the standard diagnostic procedures (which may or may not also include K-ADOS-2). Second, the ratios of ASD-to-non-ASD and male-to-female participants were unbalanced for some modules and developmental cells in our sample. For instance, in Module 4, 93.2% of the 192 participants with ASD were male, while 38.0% of the 50 participants without ASD were male. We recommend that future iterations of the study recruit a balanced number of participants in terms of diagnostic status and gender.

As more studies are reporting sex differences in symptom presentation (e.g., fewer RRBs in female individuals), which may be contributing to sex biases in diagnostic tools and practices [72], future studies should examine if there are sex differences in the validity of and symptom presentations captured by the K-ADOS-2. Third, we calculated the nonverbal mental age of some participants using VMI and Leiter International Performance Scale. However, we did not collect the nonverbal mental age of all participants because this study is a secondary analysis of pooled data and many participants were not able to measure properly due to a lack of cooperation and functional level. We retrieved available data to see the patterns of nonverbal mental ages of participants in Toddler Module and Module 1, but few participants with nonverbal mental ages lower than 15 and 12 could have been included in the analysis of Module 1 and Toddler Module, respectively. Fourth, the discriminant validity of the instrument (ASD vs. OD) should be interpreted with caution because only a small number of participants with OD were included in each developmental cell and module, and the proportion of participants without ASD may have been over-represented in this study, particularly in Modules 1–4.

Fifth, our information about the OD group was limited. We did not conduct additional or follow-up assessments to verify whether participants categorized as having OD actually have a clinical diagnosis of developmental disabilities or have comorbid disorders such as ADHD, anxiety, or obsessive–compulsive disorder (OCD). Due to missing FSIQ and VABS data, some participants without ASD who may have been categorized as the OD were not categorized as such. Sixth, while parents who responded to the VABS may not have accurately answered the questions, perhaps due to misinterpretation of the items, we did not verify the accuracy of the VABS, which was used to categorize OD, by triangulating the results with other instruments measuring adaptive skills (e.g., survey interview form of the VABS). Future studies should utilize a larger and more balanced sample including participants with a confirmed diagnosis of developmental delays or intellectual disabilities or with frequently occurring comorbid disorders (e.g., ADHD or OCD) to confirm the validity of the K-ADOS-2 in differentiating ASD from OD.

## Conclusions

This study demonstrates that K-ADOS-2 is a valid and reliable instrument for diagnosing ASD based on its sensitivity, specificity, AUC, PPV, NPV, k value, Cronbach’s alpha, and moderate agreement with existing ASD diagnostic instruments. To our knowledge, this study is the first to examine the validity and reliability of all modules and developmental cells of the K-ADOS-2. We recommend that future studies should compare K-ADOS-2 scores with best-estimate clinical diagnoses made using independent administration of standard diagnostic procedures, as well as include balanced numbers of participants in terms of gender and diagnostic status. Further, we suggest the need for studies recruiting larger samples and participants with formal diagnoses of developmental disabilities.

## Supplementary Information

**Additional file 1**.** Table S1**. Participant Characteristics by Developmental Cell.** Table S2**. Characteristics of Participants with Other Developmental (OD) Disabilities.** Table S3**. Sensitivity, Specificity, Positive Predictive Value (PPV), Negative Predictive Value (NPV), AUC, and Cohen’s Kappa value of Toddler Module based on the Mild-Moderate Concern Range of Esler et al. (2015).** Table S4**. Sensitivity, Specificity, AUC, PPV, NPV, and Cohen’s Kappa Between ASD and non-ASD Based on Autism Cut-off Criteria for Modules 1-4 and Moderate-Severe Concern Range for Toddler Module.** Table S5**. Sensitivity, Specificity, AUC, PPV, NPV, and Cohen’s Kappa Between ASD and OD Based on Autism Cut-off Criteria for Modules 1-4 and Moderate-Severe Concern Range for Toddler Module.** Table S6**. Agreement with Existing Instrument Based on Autism Cut-off Criteria for Modules 1-4 and Moderate-Severe Concern Range for Toddler Module.

## Data Availability

The datasets generated and/or analyzed during the current study are not publicly available because we do not have the permission from the IRB to share or make the unidentified participant information available online and did not receive the consent from the participants but are available from the corresponding author on reasonable request.

## Footnotes

1 We included the data from participants who were younger than 30 months when they received Module 1 (n = 10) if these data were collected prior to the publication of the Toddler Module in Korean.

2 We conducted a set of independent t-tests comparing the available subscores of the Child Behavior Checklist (CBCL) scores of participants with OD and participants without ASD and not categorized as OD (i.e., typically developing; TD) to provide information on potential comorbid conditions of the OD group. These analyses included 71 participants with OD (57% of all participants with OD), and 229 TD participants (40.0% of TD participants). Participants with OD scored significantly higher on all Syndrome Subscales except for in Social Problem Subscales when compared to the TD participants (all *p*s < .05).

3 We conducted a set of sensitivity tests excluding the participants categorized as OD, and the changes in values of sensitivity, specificity, AUC, PPV, NPV, *k* values, and α were minimal (i.e., all changes of sensitivity, specificity, PPV, and NPV were in the tenth digits, and all changes of AUC, *k*, and α were in hundredths).
