# Supplementary material for: Diagnostic validity of Autism Diagnostic Observation Schedule, second edition (K-ADOS-2) in the Korean population
Source: Mol Autism. 2022 Jun 30;13:30. doi: 10.1186/s13229-022-00506-5 (PMC9245227; doi:10.1186/s13229-022-00506-5)
Supplement: Supplementary file 1 — Additional file 1. Table S1. Participant Characteristics by Developmental Cell. Table S2. Characteristics of Participants with Other Developmental (OD) Disabilities. Table S3. Sensitivity, Specificity, Positive Predictive Value (PPV), Negative Predictive Value (NPV), AUC, and Cohen’s Kappa value of Toddler Module based on the Mild-Moderate Concern Range of Esler et al. (2015). Table S4. Sensitivity, Specificity, AUC, PPV, NPV, and Cohen’s Kappa Between ASD and non-ASD Based on Autism Cut-off Criteria for Modules 1-4 and Moderate-Severe Concern Range for Toddler Module. Table S5. Sensitivity, Specificity, AUC, PPV, NPV, and Cohen’s Kappa Between ASD and OD Based on Autism Cut-off Criteria for Modules 1-4 and Moderate-Severe Concern Range for Toddler Module. Table S6. Agreement with Existing Instrument Based on Autism Cut-off Criteria for Modules 1-4 and Moderate-Severe Concern Range for Toddler Module. [file 13229_2022_506_MOESM1_ESM.docx]

Supplementary Information

Table S1

*Participant Characteristics by Developmental Cell*

|  | | Module T(12‐20/NV21‐30) | | | Module T(21‐30SW) | | | Module 1 (NW) | | | Module 1 (SW) | | | Module 2 (< 5 yo) | | | Module 2 (>5 yo) | | |
| --- | --- | --- | --- | --- | --- | --- | --- | --- | --- | --- | --- | --- | --- | --- | --- | --- | --- | --- | --- |
|  |  | ASD  Mean (SD) | Non-ASD Mean (SD) | *t* | ASD  Mean (SD) | Non-ASD Mean (SD) | *t* | ASD  Mean (SD) | Non-ASD Mean (SD) | *t* | ASD  Mean (SD) | Non-ASD Mean (SD) | *t* | ASD  Mean (SD) | Non-ASD Mean (SD) | *t* | ASD  Mean (SD) | Non-ASD Mean (SD) | *t* |
| n | | 82 | 124 |  | 14 | 69 |  | 287 | 27 |  | 277 | 51 |  | 186 | 164 |  | 202 | 22 |  |
| Male (%) | | 76.8 | 57.3 |  | 35.7 | 55.1 |  | 77.7 | 63.0 |  | 82.7 | 66.7 |  | 81.2 | 62.2 |  | 79.7 | 50.0 |  |
| Age (months) | | 24.4 (4.5) | 19.2 (4.6) | -8.0** | 27.8 (2.0) | 25.9 (2.8) | -2.4* | 49.6 (20.2) | 36.6 (9.4) | -3.3* | 55.4 (23.1) | 38.5 (23.2) | -4.8** | 48.0 (7.3) | 40.6 (7.9) | -9.1** | 83.2 (33.3) | 68.1 (6.2) | -2.1** |
| FSIQ | | NA | NA | NA | NA | NA | NA | 61.4 (19.9) | NA | NA | 60.0 (16.8) | 97.0 (28.6) | 3.5** | 77.1 (20.7) | 91.7 (18.1) | 3.3** | 69.1 (21.4) | 94.0 (21.2) | 3.9** |
| ADOS-2 | SA^a^ | 7.9 (1.8) | 2.5 (1.7) | -22.3** | 7.4 (2.1) | 2.4 (1.4) | -8.7** | 7.2 (1.8) | 2.3 (.9) | -24.6** | 7.7 (1.7) | 2.9 (1.7) | -17.9** | 7.7 (1.6) | 2.5 (1.5) | -31.2** | 7.6 (1.5) | 2.6 (1.9) | -14.3** |
|  | RRB^a^ | 4.8 (2.4) | 1.9 (1.7) | -10.3** | 5.2 (2.4) | 1.6 (1.5) | -5.4** | 6.3 (2.2) | 2.1 (2.1) | -9.6** | 5.9 (2.3) | 1.8 (1.7) | -11.7** | 5.7 (2.4) | 2.0 (1.6) | -17.3** | 5.6 (2.2) | 1.6 (1.4) | -8.5** |
|  | Total^a^ | 7.0 (1.8) | 2.2 (1.2) | -21.5** | 7.4 (2.0) | 1.7 (1.3) | -10.2** | 6.7 (1.7) | 1.5 (.9) | -25.5** | 7.1 (1.6) | 2.2 (1.5) | -19.7** | 7.0 (1.6) | 1.7 (1.1) | -36.9** | 7.0 (1.4) | 2.1 (1.8) | -15.2** |
| ADI-R | SI | 15.1 (5.0) | 4.4 (3.3) | -18.5** | 15.3 (4.8) | 3.9 (3.0) | -11.5** | 19.8 (5.7) | 6.1 (4.3) | -12.2** | 19.2 (6.8) | 5.2 (4.4) | -14.3** | 15.0 (4.8) | 2.5 (2.5) | -29.8** | 21.3 (5.3) | 4.2 (3.9) | -14.3** |
|  | C | 1.8 (4.9) | .4 (0.9) | -1.0 | 13.4 (6.3) | 1.7 (1.8) | -8.7** | 10.5 (5.7) | 3.4 (1.2) | -3.5** | 13.1 (4.7) | 3.8 (3.5) | -11.6** | 13.1 (4.0) | 1.7 (1.7) | -33.6** | 16.3 (4.3) | 2.5 (2.6) | -14.5** |
|  | RRB | 3.4 (2.0) | 1.0 (1.2) | -10.9** | 3.9 (2.8) | .7 (.9) | -7.7** | 4.8 (2.1) | 1.4 (1.4) | -8.2** | 4.8 (2.2) | 1.3 (1.5) | -10.9** | 5.7 (2.7) | .9 (1.3) | -20.5** | 5.9 (2.8) | .8 (1.1) | -8.1** |
| K-CARS | | 31.5 (5.1) | 17.6 (3.7) | -22.5** | 32.1 (4.5) | 17.3 (3.1) | -15.1** | 36.1 (5.9) | 19.8 (3.9) | -13.3** | 32.8 (5.2) | 18.5 (2.9) | -16.5** | 32.0 (5.1) | 17.0 (2.2) | -30.1** | 33.3 (4.8) | 21.2 (5.3) | -7.6** |
| SCQ | | 15.3 (4.6) | 8.5 (5.2) | -6.6** | 14.8 (4.1) | 6.6 (5.3) | -4.0** | 17.6 (6.2) | 5.8 (3.6) | -5.7** | 16.2  (6.9) | 6.1 (6.1) | -7.0** | 13.7 (6.6) | 2.80 (2.8) | -14.1** | 13.8 (7.4) | 4.0 (5.7) | -5.8** |
| SRS | | 63.7 (16.5) | 49.2 (7.4) | -7.8** | 68.8 (15.2) | 46.3 (8.9) | -7.0** | 90.8 (30.7) | 49.9 (14.9) | -6.2** | 87.7 (30.2) | 43.8 (20.9) | -8.7** | 79.1 (27.7) | 35.1 (17.3) | -17.0** | 86.9 (29.5) | 37.0 (31.2) | -7.2** |

*Note*. **p*<.05, ***p*<.01. ASD, autism spectrum disorder; *t*, t-value from independent sample t-test between ASD and non-ASD; 12‐20/NV21‐30, 12-20 months toddlers/nonverbal 21-30 months toddlers; 21‐30 SW, 21-30 months toddlers with some words; NW, no words; SW, some words; yo, years old; FSIQ, full-scale intelligence quotient; ADOS-2, Autism Diagnostic Observation Schedule-2; SA, Social Affect; RRB, Restricted Repetitive Behavior; ADI-R, Autism Diagnostic Interview-Revised; SI, Social Interaction; C, Communication Total; RRB, Restricted Repetitive; K-CARS, Korean Child Autism Rating Scale; SCQ, Social Communication Questionnaire; SRS, Social Responsiveness Scale. ^a^ = reported in calibrated severity scores.

Table S2

*Characteristics of Participants with Other Developmental (OD) Disabilities*

|  | | Total | Module T | Module T (12‐20/NV21‐30) | Module T (21‐30SW) | Module 1 | Module 1 (NW) | Module 1 (SW) | Module 2 | Module 2 (< 5yo) | Module 2 (>5 yo) | Module 3 | Module 4 |
| --- | --- | --- | --- | --- | --- | --- | --- | --- | --- | --- | --- | --- | --- |
| n | | 123 | 22 | 15 | 7 | 27 | 12 | 15 | 26 | 19 | 7 | 39 | 9 |
| % Male | | 62.6** | 72.7 | 73.3 | 71.4 | 63.0* | 75.0 | 53.3** | 50.0** | 63.2 | 14.3** | 61.5** | 77.8 |
| Age (months) | | 79.3 (65.4) | 24.5 (4.6) | 23.9 (5.2) | 25.7 (2.7) | 44.4 (32.1) | 39.2 (13.6) | 48.6 (41.5) | 50.2 (14.2)* | 43.0 (7.8)* | 69.9 (6.7) | 116.1 (34.1) | 243.0 (61.3) |
| FSIQ | | 82.1 (18.8) | NA | NA | NA | 81.0 (NA) | NA | 81.0 (NA) | 71.9 (15.0) | 74.6 (16.7) | 66.0 (9.1) | 87.7 (19.0) | 73.0 (15.4) ** |
| ADOS-2 | SA^a^ | 3.4 (2.3)*** | 3.9 (2.6)** | 3.9 (3.0)** | 3.7 (1.6)** | 2.9 (1.5)** | 2.7 (.9)** | 3.1 (1.9)** | 2.6 (1.5)** | 2.6 (1.4)** | 2.6 (1.9)** | 4.1 (2.7)** | 3.0 (2.2)** |
|  | RRB^a^ | 1.8 (1.6)** | 2.1 (1.8)** | 2.6 (2.0)** | 1.00 (0)** | 1.7 (1.6)** | 1.7 (1.7)** | 1.6 (1.6)** | 2.4 (1.9)** | 2.7 (2.0)** | 1.4 (1.1)** | 1.2 (0.8)** | 2.0 (2.0)* |
|  | Total^a^ | 2.5 (1.9)** | 3.1 (2.1)** | 3.2 (2.2)** | 2.9 (2.0)** | 2.1 (1.6)** | 1.8 (1.2)** | 2.4 (1.9)** | 1.9 (1.0)** | 1.8 (1.0)** | 1.9 (1.2)** | 3.0 (2.3)** | 2.1 (1.3)** |
| ADI-R | SI | 5.5 (4.7)** | 8.4 (4.5)** | 8.4 (4.9)** | 8.3 (3.9)* | 7.2 (5.4)** | 7.6 (5.4)** | 6.9 (5.6)** | 3.2 (3.4)** | 2.6 (3.0)** | 4.9 (4.1)** | 4.5 (3.9)** | 4.0 (4.7)** |
|  | C | 3.0 (3.2)** | NA | NA | NA | 4.3 (3.3)** | 3 (NA) | 4.5 (3.6)** | 2.5 (2.3)** | 2.3 (2.0)** | 2.9 (3.2)** | 3.1 (3.6)** | 3.1 (3.7)** |
|  | RRB | 1.2 (1.3)** | 1.9 (1.3)** | 1.9 (1.4)* | 1.9 (.9) | 1.4 (1.5)** | 1.5 (1.6)** | 1.4 (1.6)** | 1.0 (.9)** | .8 (.8)** | 1.4 (1.3)** | .8 (1.3)** | 1.4 (1.7)** |
| K-CARS | | 20.0 (4.5)** | 22.5 (5.8)** | 22.5 (6.8)** | 22.4 (3.3)** | 20.1 (3.5)** | 20.9 (3.7)** | 19.1 (3.1)** | 18.0 (2.6)** | 17.1 (1.9)** | 21.0 (2.7)** | 19.7 (4.2)** | 17.0 (2.2)** |
| SCQ | | 6.0 (6.1)** | 11.8 (4.8)* | 11.3 (4.2)* | 12.8 (6.2) | 7.6 (8.1)** | 4.8 (4.3)** | 9.1 (9.4) | 3.2 (3.1)** | 2.9 (2.8)** | 3.7 (3.8)** | 4.6 (5.5)** | 8.4 (5.5) |
| SRS | | 45.7 (26.8)** | 57.3 (8.2) | 56.3 (8.4) | 59.5 (8.0) | 51.3 (26.3)** | 55.9 (21.9)* | 48.1 (29.4)** | 37.4 (20.4)** | 37.5 (20.0)** | 37.3 (23.8)** | 37.2 (27.3)** | 67.8 (44.0)* |

*Note*. *** Significantly different from the means of ASD sample, *p* < .05, ** *p* < .01. *t*, t-value from independent sample t-test between autism and non-autism; 12‐20/NV21‐30, 12-20 months toddlers/nonverbal 21-30 months toddlers; 21‐30 SW, 21-30 months toddlers with some words; NW, no words; SW, some words; yo, years old; FSIQ, full-scale intelligence quotient; ADOS-2, Autism Diagnostic Observation Schedule-2; SA, Social Affect; RRB, Restricted Repetitive Behavior; ADI-R, Autism Diagnostic Interview-Revised; SI, Social Interaction; C, Communication Total; RRB, Restricted Repetitive; K-CARS, Korean Child Autism Rating Scale; SCQ, Social Communication Questionnaire; SRS, Social Responsiveness Scale. ^a^ = reported in calibrated severity scores.

Table S3

*Sensitivity, Specificity, Positive Predictive Value (PPV), Negative Predictive Value (NPV), AUC, and Cohen’s Kappa value of Toddler Module based on the Mild-Moderate Concern Range of Esler et al. (2015)*

|  | ASD  n | Non-ASD n | Sensitivity (%) | Specificity  (%) | AUC | PPV  (%) | NPV  (%) | Cohen’s Kappa  (p-value) |
| --- | --- | --- | --- | --- | --- | --- | --- | --- |
| Toddler Module  (12‐20/NV21‐30) | 82 | 124 | 100.0 | 93.5 | .97 | 91.1 | 100.0 | .92**  (*p*<.001) |
| Toddler Module  (V21‐ 30SW) | 14 | 69 | 100.0 | 94.2 | .97 | 77.8 | 100.0 | .85**  (*p*<.001) |

*Notes.* 12‐20/NV21‐30, 12-20 months toddlers/nonverbal 21-30 months toddlers; 21‐30 SW, 21-30 months toddlers with some words;

Table S4

*Sensitivity, Specificity, AUC, PPV, NPV, and Cohen’s Kappa Between ASD and non-ASD Based on Autism Cut-off Criteria for Modules 1-4 and Moderate-Severe Concern Range for Toddler Module*

|  | n | Sensitivity (%) | Specificity  (%) | AUC | PPV  (%) | NPV  (%) | Cohen’s Kappa  (p-value) |
| --- | --- | --- | --- | --- | --- | --- | --- |
| Total | 2158 | 86.6 | 96.2 | .91 | 98.0 | 76.9 | .78**  (*p*<.001) |
| Toddler Module | 289 | 87.5 | 95.9 | .92 | 91.3 | 93.9 | .84**  (*p*<.001) |
| Toddler Module  (12‐20/NV21‐30) | 206 | 85.4 | 96.8 | .91 | 94.6 | 90.9 | .84**  (*p*<.001) |
| Toddler Module  (V21‐ 30SW) | 83 | 100 | 94.2 | .97 | 77.8 | 100 | .85**  (*p*<.001) |
| Module 1 Total | 642 | 83.7 | 97.4 | .91 | 99.6 | 45.1 | .54**  (*p*<.001) |
| Module 1  (NW) | 314 | 81.2 | 100.0 | .91 | 100.0 | 33.3 | .43**  (*p*<.001) |
| Module 1  (SW) | 328 | 86.3 | 96.1 | .91 | 99.2 | 56.3 | .64**  (*p*<.001) |
| Module 2 Total | 574 | 89.7 | 97.3 | .94 | 98.6 | 81.9 | .83**  (*p*<.001) |
| Module 2  (< 5 yo) | 350 | 84.9 | 98.8 | .92 | 98.8 | 85.3 | .83**  (*p*<.001) |
| Module 2  (> 5 yo) | 224 | 94.1 | 86.4 | .90 | 98.4 | 61.3 | .68**  (*p*<.001) |
| Module 3 | 411 | 87.6 | 93.8 | .91 | 94.9 | 85.2 | .80**  (*p*<.001) |
| Module 4 | 242 | 87.0 | 100.0 | .94 | 100 | 66.7 | .73**  (*p*<.001) |

*Note*. **p*<.05, ***p*<.01. AUC, area under curve; PPV, positive predictive value; NPV, negative predictive value; ASD, autism spectrum disorder; 12‐20/NV21‐30, 12-20 months toddlers/nonverbal 21-30 months toddlers; 21‐30 SW, 21-30 months toddlers with some words; NW, no words; SW, some words; yo, years old.

Table S5

*Sensitivity, Specificity, AUC, PPV, NPV, and Cohen’s Kappa Between ASD and OD Based on Autism Cut-off Criteria for Modules 1-4 and Moderate-Severe Concern Range for Toddler Module*

|  | N of ASD | N of OD | Sensitivity (%) | Specificity  (%) | AUC | PPV  (%) | NPV  (%) | Cohen’s Kappa  (p-value) |
| --- | --- | --- | --- | --- | --- | --- | --- | --- |
| Total | 1473 | 123 | 86.6 | 89.4 | .88 | 99.0 | 35.7 | .45**  (*p*<.001) |
| Toddler Module | 96 | 22 | 87.5 | 81.8 | .85 | 95.5 | 60 | .60**  (*p*<.001) |
| Toddler Module  (12‐20/NV21‐30) | 82 | 15 | 85.4 | 80.0 | .83 | 95.9 | z | .53**  (*p*<.001) |
| Toddler Module  (21‐30SW) | 14 | 7 | 100 | 85.7 | .93 | 93.3 | 100 | .89**  (*p*<.001) |
| Module 1 | 564 | 27 | 83.7 | 92.6 | .88 | 99.6 | 21.4 | .29**  (*p*<.001) |
| Module 1  (NW) | 287 | 12 | 81.2 | 100.0 | .91 | 100.0 | 18.2 | .26**  (*p*<.001) |
| Module 1  (SW) | 277 | 15 | 86.3 | 84.7 | .87 | 99.2 | 25.5 | .34**  (*p*<.001) |
| Module 2 | 388 | 26 | 89.7 | 100.0 | .95 | 100.0 | 39.4 | .52**  (*p*<.001) |
| Module 2  (< 5 yo) | 186 | 19 | 84.9 | 100.0 | .93 | 100.0 | 40.4 | .51**  (*p*<.001) |
| Module 2  (> 5 yo) | 202 | 7 | 94.1 | 100.0 | .97 | 100.0 | 36.8 | .51**  (*p*<.001) |
| Module 3 | 233 | 39 | 87.6 | 82.1 | .85 | 96.7 | 52.5 | .56**  (*p*<.001) |
| Module 4 | 192 | 9 | 87.0 | 100.0 | .94 | 100.0 | 26.5 | .37**  (*p*<.001) |

*Note*. **p*<.05, ***p*<.01. AUC, area under curve; PPV, positive predictive value; NPV, negative predictive value; ASD, autism spectrum disorder; OD, other developmental disabilities; 12‐20/NV21‐30, 12-20 months toddlers/nonverbal 21-30 months toddlers; 21‐30 SW, 21-30 months toddlers with some words; NW, no words; SW, some words; yo, years old.

Table S6

*Agreement with Existing Instrument Based on Autism Cut-off Criteria for Modules 1-4 and Moderate-Severe Concern Range for Toddler Module*

|  | Kappa values | | | | |
| --- | --- | --- | --- | --- | --- |
|  | ADI-R | SCQ | SRS | K-CARS (cut-off 24) | K-CARS (cut-off 28) |
| Total Modules | .61* | .51* | .58* | .58* | .56* |
| Toddler Module | .58* | .49* | .57* | .86* | .83* |
| Toddler Module  (12‐20/NV21‐30) | .59* | .48* | .58* | .85* | .83* |
| Toddler Module  (21‐30SW) | .49* | .47* | .51* | .90* | .81* |
| Module 1 | .37* | .42* | .44* | .45* | .49* |
| Module 1 (NW) | .31* | .34* | .37* | .38* | .52* |
| Module 1 (SW) | .42* | .49* | .49* | .52* | .46* |
| Module 2 | .65* | .50* | .60* | .62* | .58* |
| Module 2 (< 5 yo) | .65* | .49* | .61* | .61* | .58* |
| Module 2 (> 5 yo) | .45* | .35* | .39* | .29* | .27* |
| Module 3 | .71* | .52* | .58* | .40* | .39* |
| Module 4 | .51* | .45* | .49* | .22* | .20* |

*Note*. **p*<.001. 12‐20/NV21‐30, 12-20 months toddlers/nonverbal 21-30 months toddlers; 21‐30SW, 21-30 months toddlers with some words; NW, no words; SW, some words; yo, years old; ADI-R, Autism Diagnostic Interview-Revised; SCQ, Social Communication Questionnaire; SRS, Social Responsiveness Scale; K-CARS, Korean Child Autism Rating Scale
